# Comparison of Performance, Egg Quality, and Yolk Fatty Acid Profile in Two Turkish Genotypes (Atak-S and Atabey) in a Free-Range System

**DOI:** 10.3390/ani11051458

**Published:** 2021-05-19

**Authors:** Arda Sözcü, Aydın İpek, Züleyha Oguz, Stefan Gunnarsson, Anja B. Riber

**Affiliations:** 1 Department of Animal Science, Faculty of Agriculture, Bursa Uludağ University, Bursa 16059, Turkey; aipek@uludag.edu.tr; 2 Poultry Research Institute, Republic of Turkey Ministry of Agriculture and Forestry, Ankara 06560, Turkey; zuleyha.oguz@tarimorman.gov.tr; 3 Department of Animal Environment and Health, Swedish University of Agricultural Sciences (SLU), 53223 Skara, Sweden; stefan.gunnarsson@slu.se; 4 Department of Animal Science, Aarhus University, 8830 Aarhus, Denmark; anja.riber@anis.au.dk

**Keywords:** egg production, fatty acids, free range, genotype, yolk color, poultry

## Abstract

**Simple Summary:**

In recent years, consumers have shown increased interest in healthy and safe food produced under improved animal welfare standards. Therefore, production systems proving outdoor access have gained popularity, increasing the need for knowledge on genotypes suitable for free-range systems. This study aimed to investigate the suitability of two Turkish layer genotypes, Atak-S (brown) and Atabey (white), in a free-range system. We evaluated laying performance, egg quality parameters, and yolk fatty acid profile. The egg production was higher in Atabey than Atak-S, whereas the eggs from Atak-S hens tended to be heavier and had a stronger shell structure than eggs from Atabey hens. Furthermore, eggs from Atabey hens had improved egg yolk and albumen content compared to eggs from Atak-S hens. The total saturated fatty acid content in yolk was higher in Atabey eggs than in Atak-S eggs at 56 weeks of age, whereas a higher yolk color score was observed in Atak-S eggs than in Atabey eggs. Our results could help free-range egg producers to improve production, as well as satisfy consumer demands regarding egg quality in organic production.

**Abstract:**

Consumer interest in buying eggs from animal welfare-friendly systems with outdoor access is increasing, leading to an increase in the need for knowledge on genotypes suitable for free-range systems. Two Turkish laying hen genotypes, Atak-S (brown, *n* = 210) and Atabey (white, *n* = 210), were reared in a free-range system from 19–72 weeks of age, and their suitability for the system was assessed based on laying performance, egg quality, and yolk fatty acid profile. Mean hen-day and hen-housed egg production were found to be higher in Atabey than Atak-S (*p* < 0.01). The brown eggs from Atak-S hens tended to be heavier than the white eggs from Atabey hens (*p* < 0.01). Brown eggs obtained from Atak-S hens had a stronger shell structure (*p* < 0.01), while white eggs from Atabey hens had higher mean yolk index, albumen index, and Haugh unit than brown eggs (*p* < 0.05). At 56 weeks of age, total saturated fatty acid content in yolk was higher in white eggs than in brown eggs (*p* < 0.01). These findings related to genotype could help free-range egg producers in their choices for more profitable production and for meeting consumer demands on egg quality and egg yolk fatty acid levels.

## 1. Introduction

Egg production is a major and significant component of animal production in Turkey, due to its high economic contribution (1.7 billion egg production in 2020) and its adaptability to new sectoral developments and consumer demands [1]. Consumers are now demanding food produced within organic production systems, with stricter safety rules and nature protection concepts [2]. This is resulting in alternative production systems with better regard for animal rights and welfare issues in poultry production world-wide [3]. It is known that intensive production systems can affect egg quality parameters due to high levels of stress and poor welfare of laying hens [4,5]. Therefore, free-range and organic production systems have gained popularity, as they can decrease the stressful conditions in intensive poultry systems and increase the comfort and improve the welfare status and behavioral patterns of commercial birds [6].

Free-range and organic systems both provide laying hens with access to outdoor areas with more physical activities, natural light, and sunshine and scope to exhibit natural behaviors, such as foraging, dust bathing, perching, nesting, preening, and pecking [7]. However, previous studies comparing intensive and alternative systems using commercial layer hybrids with high yield performance have shown that productivity, egg quality, and hen health and welfare status can be lower in alternative systems with outdoor access [8]. Problems observed in free-range and organic systems include increased morbidity due to footpad dermatitis or bumblefoot, lower feed efficiency, and increased mortality due to injurious pecking or predation [8]. The negative issues reported for high-yielding hybrids in production systems with outdoor access could be because these genotypes are more suitable for production under highly controlled conditions [9]. They may, therefore, have difficulty adapting to the less controlled conditions in outdoor areas and less equilibrated rations in free-range systems [10].

To alleviate welfare, health, and performance loss problems, EU Regulation 1804/99 [11] and the final recommendation of the Network for Animal Health and Welfare in Organic Agriculture [12] suggest the use of native genotypes, because of their robustness and rusticity [10,13] and adaptability to different geographical regions and local climate conditions. The use of native breeds could ensure vitality and resistance to disease, as well as protecting biodiversity [14].

Free-range systems and organic egg production are gaining importance in Turkey due to their potential to produce healthier eggs; protect the comfort, health, and welfare status of laying hens; and improve environmental protection aspects. Turkish egg production involves three main types of production systems, with 78.3% of all eggs in 2018 produced in conventional systems (unenriched cages and enriched cage systems) and 21.7% in alternative systems (19.8% in free-range systems and 1.9% in organic systems) [15].

The present study was part of the FreeBirds project which is aimed is to develop more effective husbandry practices in organic poultry production to make the chickens be more outdoor, in accordance with the intentions of the organic concept. The Turkish team investigated the suitability of two local egg-type genotypes bred by the Turkish Poultry Research Institute. Hens of Atak-S (brown egg genotype) and Atabey (white egg genotype) were evaluated in a free-range system under Turkish field conditions for their production performance. There is little information in the literature on the productivity of these two Turkish genotypes during the whole production period (19–72 weeks of age) in a free-range system. The aim of the study was thus to obtain performance data on the two genotypes of layers, Atak-S and Atabey, kept in a free-range system.

A further aim was to develop management tools and pasturing strategies to improve the production performance of local genotypes kept under field conditions in free-range systems and formulate some practical proposals for organic production. Specific objectives of the present study were to determine productivity, egg quality, and egg yolk fatty acid profiles for the Atak-S and Atabey laying hen genotypes and then assess the suitability and adaptability of the two genotypes for pasturing in a free-range system.

## 2. Materials and Methods

### 2.1. Animals and Housing

A total of 420 laying hens of two local layer genotypes, 210 Atak-S (brown) and 210 Atabey (white), were studied between 19 and 72 weeks of age. The experimental design consisted of the two genotypes with three subgroups (*n* = 3 pens/genotype, 70 hens/pen), which were taken as replicates of each genotype. The hens were individually weighed on a digital scale with precision ±0.1 g to determine body weight (BW). Then, the hens were randomly allocated to the pens, all of which had dimensions 3 m × 7 m.

All hens were kept in a free-range system that was generated according to the minimum standards in EU Directive 1999/74/EC [16]. In compliance with these regulations, the free-range system had an indoor area and outdoor pasture area. The indoor floor was covered with wood shavings as litter material. Circular plastic feeders (6.3 cm feeder area/hen) and plastic bell drinkers (4.5 cm drinker area/hen) were provided in the indoor area. The indoor area was also enriched with perches (18 cm perch length/hen) and nesting boxes (3.5 hens/nesting box). The stocking density was 3.33 hens per m^2^ in the inside area. The pasture area (350 m^2^/pen) was enclosed by wire fences to keep out predators and contained a shelter. The stocking density was 5 hens per m^2^ in the pasture area. The lighting regime in the house was gradually increased, by 1 h per week, from 14 h per 24 h period at 19 weeks to 16 h per 24 h period from 20 weeks to 72 weeks of age.

**Figure 1:**
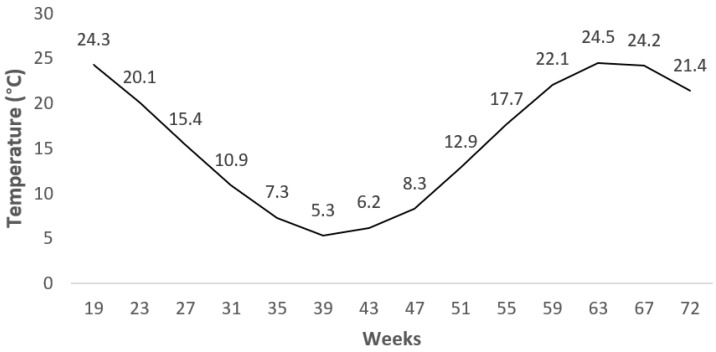
Mean value of temperature during experimental period.

**Figure 2:**
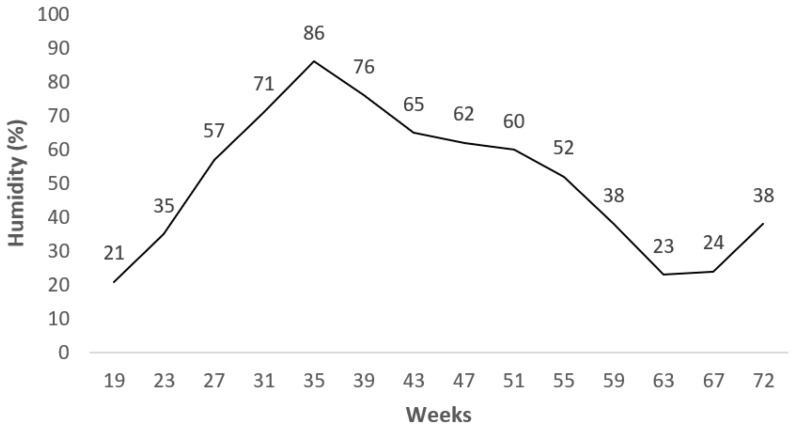
Mean value of humidity during experimental period.

A standard layer diet for free-range systems was used between 19 and 40 weeks of age as the phase I diet (17.86% crude protein (CP), 2750 metabolizable energy (ME) kcal/kg), and a different standard diet was used between 41 and 72 weeks of age as the phase II diet (16.45% CP, 2800 ME kcal/kg) (Table 1). The nutrient level in the diets was analyzed according to [17]. Feed and water were offered ad libitum throughout the experiment. The pasture area was cultivated with 60% perennial ryegrass (*Lolium perenne*), 30% alfalfa (*Medicago sativa*), and 10% white clover (*Trifolium repens*). The hens could supplement their diets with vegetation and living small creatures (insects, arthropods, etc.) in the foraging area. During the experimental period, the climate conditions were monitored, and the average values of the temperature and humidity are shown in Figure 1 and Figure 2.

### 2.2. Data Collection

To determine mean BW, hens (20% from each pen) were individually weighed weekly from 18 to 72 weeks of age. Daily feed intake (DFI) and egg weight (EW) were recorded on a weekly basis. The pens were monitored daily for egg production (EP) and mortality until the end of the experiment. Egg production was calculated by dividing the number of eggs daily collected by the number of hens on the same day. The efficiency of feed utilization (FCR) was calculated as the ratio between weekly feed intake and weekly EP multiplied by EW. Daily collected eggs were classified according to shell surface cleanliness into three categories, namely very dirty (shell surface > 1 cm^2^ dirty), dirty (shell surface < 1 cm^2^ dirty), and clean (no dirt on the shell), and as cracked eggs or floor eggs. Percentage values of the categories were calculated by dividing the number of dirty/cracked/floor eggs by the total number of eggs. Mean BW, DFI, EP, EW, and FCR were calculated for each 6-week period between 19 and 72 weeks of age.

A total of 30 eggs from each genotype were randomly sampled to define external and internal egg quality parameters at 24, 40, 56, and 72 weeks of age. The measurements were performed 24 h after the eggs were laid. After weighing the eggs with ±0.01 g precision, the length and width of the eggs were measured by using a digital caliper with 0.01 mm precision (Mitutoyo, 300 mm, Neuss, Germany). The measured values were used to calculate the egg shape index with a formula of (egg width/egg length) × 100 [18]. Eggshell breaking strength (kg/cm^2^) was determined by using an eggshell force reader machine (Egg Force Reader, Orka Food Technology, Israel). The eggs were broken to obtain the albumen and yolk, and then the yolk weight was measured with ±0.01 g precision. The eggshells were cleaned by washing process and then put in an oven at 105 °C (Nüve FN-500, Ankara, Turkey) for drying process for 24 h. Then, the eggshell weight was determined with ±0.01 g precision. Albumen weight was calculated by subtracting yolk and shell weight from total egg weight. The ratios of albumen, eggshell, and yolk were given as a percentage of EW. Eggshell thickness was measured at three different points of the eggshell, specifically air cell, sharp end, and equator region, by using a digital caliper with ±0.01 mm precision. The eggshell thickness was given as the average of three values measured for these points.

To calculate the yolk index (YI), albumen index (AI), and Haugh unit (HU), yolk diameter (YD), albumen length (AL), and albumen width (AW) were determined by using a digital caliper with ±0.01 mm precision (Mitutoyo, 300 mm, Neuss, Germany). Albumen height (AH) and yolk height (YH) were measured by using a tripod micrometer. Egg yolk index, albumen index, and Haugh unit were calculated using the formulas given by Funk, Heiman and Carver, and Haugh [19,20,21], respectively:

YI = (YH/YD) × 100

AI = (AH/(AL + AW)/2)) × 100

HU = 100 × log (AH + 7.57 − 1.7 × EW0.37)

Yolk color was determined with a Roche yolk color fan with a 15-point scale (Roche Ltd., Basel, Switzerland), according to the pigmentation degree from the lightest (score 1) to the darkest color (score 15).

At 56 weeks of age, 12 yolk and albumen samples were randomly selected from each genotype and analyzed for dry matter (method number 934.01), ash (method number 942.05), protein (method number 954.01), and lipid (method number 920.39) content, according to AOAC (2006) [17].

To determine the fatty acid profile, 12 yolk samples from each genotype were randomly obtained at 56 weeks of age. Yolk fatty acid analysis was performed using a gas chromatography method developed by Folch et al. [22]. The fatty acid profile of the egg yolk was expressed as a percentage of total fatty acids [23].

### 2.3. Statistical Analysis

The parametric data for performance parameters (BW, EP, DFI, EM, and FCR) for each genotype (Atak-S and Atabey) were analyzed with the mixed model procedure in the statistical analysis software SAS (version 9.4, 2012, Cary, NC, USA). A completely randomized, repeated measures design on a weekly basis was used for performance parameters, and the mean values for each parameter were calculated for consecutive 6-week periods for the whole production period. For performance parameters, egg external and internal quality parameters, and egg chemical composition, the main effects (G = effect of genotypes and A = effect of age) and the combined effect (G × A interaction) were determined. Significant differences between means were compared using the Tukey test. Analyses of percentage data were conducted after arcsine square root transformation of the data. The total mortality data were analyzed using chi-square tests to determine differences between the genotypes. The effects of genotype on egg chemical composition and yolk fatty acid profile were subjected to the *t*-test procedure in SAS (version 9.4, 2012, Cary, NC, USA). Differences were considered statistically significant at *p* ≤ 0.05.

## 3. Results

Mean egg production and egg weight for the brown (Atak-S) and white (Atabey) layer genotypes during their production life in the free-range system are presented in Table 2. A significant genotype × age interaction was found for hen-day and hen-house EP (*p* < 0.01). At 19–24 weeks of age, Atak-S hens showed higher hen-day and hen-house EP levels (56.4% and 55.4%, respectively) than Atabey hens (40.4% and 40.2%, respectively) (*p* < 0.01). However, Atabey hens reached a peak level at 25–30 weeks of age, with an EP level of 90.1% on a hen-day basis and 89.2% on a hen-house basis (*p* < 0.01). The laying performance of Atabey hens decreased to below 80% after 54 weeks of age, whereas Atak-S hens showed a rapid decline to a similar EP level after 37 weeks of age. Egg weight showed significant differences arising from genotype × age interactions.

The effects of genotype on BW, DFI, and FCR are presented in Table 3. The results indicated that as the hens aged, DFI increased in both genotypes, with a corresponding increment in BW. As expected, BW was found to be higher in Atak-S than Atabey (2087.3 g vs. 1497.4; *p* < 0.01). Atak-S hens tended to consume more feed than Atabey hens. Mean DFI was found to be higher in Atak-S (117.2 g) than in Atabey (109.8 g) (*p* < 0.01). Based on the difference in BW, DFI and FCR differed significantly between two genotypes during the whole experimental period. Higher FCR between 19 and 72 weeks of age was observed in Atabey than in Atak-S (2.48 vs. 2.54; *p* = 0.001). Mortality between 19 and 72 weeks of age was 11.0% in Atak-S and 8.1% in Atabey, although no significant difference was found (chi-square value = 0.995, *p* = 0.319).

**Figure 3:**
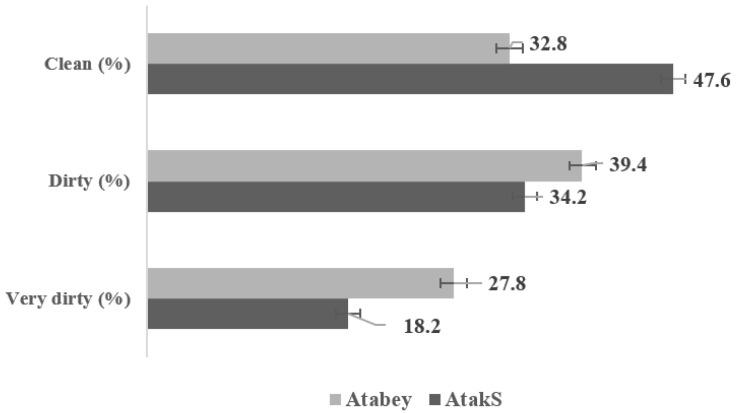
Cleanness classification of eggs from Atak-S and Atabey hens in a free-range system. Bars represent mean ± SE. (*p* < 0.01, *n* = 3 replicates per treatment group, 70 laying hens per replicate pen).

**Figure 4:**
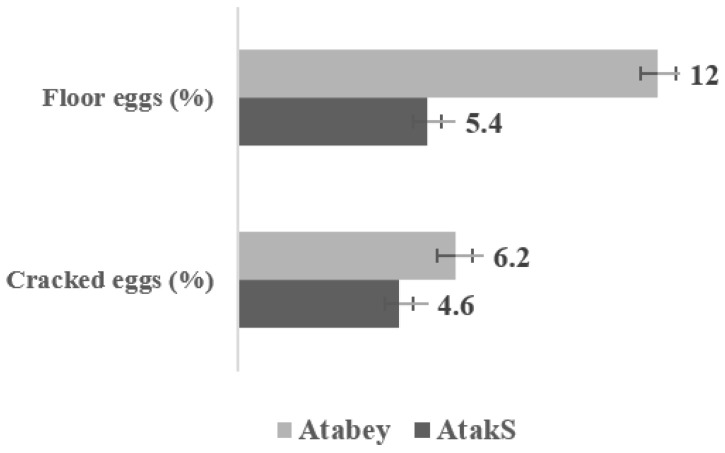
Percentage of cracked and floor eggs from Atak-S and Atabey hens in a free-range system. Bars represent mean ± SE. (*p* < 0.05, *n* = 3 replicates per treatment group, 70 laying hens per replicate pen).

The proportions of eggs with different degrees of shell cleanness differed between the genotypes (Figure 3). The proportions of very dirty and dirty eggs were found to be higher for Atabey (27.8% and 39.4% respectively) than for Atak-S (18.2% and 34.2%, respectively) (*p* < 0.001), whereas the ratio of clean eggs was higher for Atak-S than Atabey (47.6% vs. 32.8%; *p* < 0.001). Higher percentages of cracked and floor eggs were observed for Atabey (6.2% and 12.0%, respectively) than Atak-S (4.6% and 5.4%, respectively) (*p* < 0.05) (Figure 4).

An effect of genotype on egg composition was found (Table 4). The interaction (genotype × age) was significant for EW (*p* < 0.01), yolk ratio, and albumen ratio (*p* < 0.05), but not for shell ratio. Yolk ratio was found to be higher at 56 and 72 weeks of age in brown (Atak-S) eggs and at 40, 56, and 72 weeks of age in white (Atabey) eggs. The albumen ratio showed a decline with hen age for both Atak-S and Atabey (*p* < 0.01). The shell ratio was only affected by hen genotype (*p* < 0.01).

An effect of genotype on egg exterior quality parameters was also observed (Table 5). A significant interaction (genotype × age) was observed for egg shape index (*p* = 0.027) and eggshell thickness (*p* < 0.01). The highest mean value of shape index was observed for Atak-S eggs at 24 weeks of age (79.7%). For both Atak-S and Atabey eggs, eggshells tended to be thinner at 72 weeks of age (0.347 and 0.320 mm) than at earlier ages, more so for Atabey than Atak-S eggs. On the other hand, breaking strength was affected by genotype and age. Atak-S eggs had a stronger eggshell structure (3.429 g/cm^2^) than Atabey eggs (2.982 g/cm^2^) (*p* < 0.01). As hen age increased, the breaking strength of the eggs showed a deterioration, with the mean value for all hens decreasing from 3.398 g/cm^2^ at 24 weeks of age to 2.890 g/cm^2^ at 72 weeks of age (*p* < 0.01).

As regards egg interior quality parameters, genotype significantly affected yolk index (*p* = 0.026), yolk color (*p* < 0.01), albumen index (*p* < 0.01), and Haugh unit (*p* < 0.01) (Table 6). Higher mean values of yolk index, albumen index, and Haugh unit were observed for Atabey eggs (44.4%, 12.3%, and 92.4, respectively) compared with Atak-S eggs (43.1%, 10.9%, and 88.5, respectively). Yolk index had the highest mean value at 56 weeks of age (46.9%) and declined to 43.7% at 72 weeks of age (Table 6). Furthermore, higher yolk color score was observed in Atak-S eggs than in Atabey eggs (12.9 vs. 12.2; *p* < 0.01). Yolk color score was highest at 40 and 56 weeks of age and then started to decrease after 56 weeks (*p* = 0.016). On the other hand, albumen index and Haugh unit started to decrease after 40 weeks of age in both genotypes.

The effects of genotype on egg chemical composition and yolk fatty acid profile at 56 weeks of age are shown in Table 7. The dry matter, ash, and fat contents in yolk were found not to differ between two genotypes, whereas a higher content of protein of yolk was observed in Atabey eggs (*p* = 0.031). The dry matter content of albumen was significantly higher in Atabey eggs than in Atak-S eggs (88.2% vs. 86.6%; *p* = 0.009), whereas a higher protein content of albumen was observed in Atak-S eggs (13.6% vs. 11.9%; *p* = 0.009). The saturated fatty acid profile of yolk, except C15:0, was significantly affected by genotype, and the total saturated fatty acid content of yolk was found to be higher in Atabey eggs (35.4%) than in Atak-S eggs (32.1%) (*p* < 0.01). The unsaturated fatty acid profile of yolk, except C18:1c, C18:2c, and C20:3n6, was significantly affected by genotype, whereas the totals of UFAs, MUFAs, and PUFAs were found not to differ between the genotypes. Higher ratios of UFAs/SFAs, MUFAs/SFAs, and PUFAs/SFAs were observed in Atak-S eggs than in Atabey eggs (*p* < 0.01).

## 4. Discussion

This study showed that Atabey hens had a higher EP level in a free-range system than Atak-S hens. Between 19 and 24 weeks of age, Atabey hens displayed better laying performance, with approximately 3.2% and 3.8% higher hen-day and hen-house EP compared with Atak-S hens. According to the standard performance data for Atak-S and Atabey given in the management and performance guide [24], under optimum management standards in cage systems, hen-day and hen-house EP are 83.3% and 82.4%, respectively, for Atak-S, and 83.9% and 82.8%, respectively, for Atabey. This is better laying performance than observed in our free-range system and may be attributed to differences between the cage system and free-range system in terms of stress level, nutrition, physical activity level, and varying environmental conditions, especially during pasturing of laying hens in the free-range system [25]. Egg formation is an energy-requiring activity, so the observed decline in EP level could be attributable to the hens in this study spending energy for physical activity while outdoor ranging.

Hens of both genotypes produced heavier eggs with increasing age, confirming previous findings of [26] for Italian genotypes and [27] for conventional and traditional genotypes (Lohmann Brown vs. Banat Naked-Neck). In the present study on a free-range system with outdoor access, the hens tended to show reduced egg weight and EP level. This is consistent with the findings of Samiullah et al. [28], Miao et al. [29], and Campbell et al. [30]. In contrast, some studies have found no significant differences in EW for eggs obtained from free-range systems and conventional unenriched cage systems [31,32,33]. Compared with standard values given in the performance guide for the Atabey and Atak-S genotypes, lower BW of hens and higher DFI were recorded in this study. Our findings are consistent with previous results reported by Mugnai et al. [10], Lampkin [34] and Küçükyılmaz et al. [35]. Those studies attributed higher feed intake in organic systems with outdoor access to increased physical activity, including walking and foraging behaviors, which also increased the energy requirement of laying hens. The higher percentages of cracked and floor eggs observed for Atabey hens in the present study could be related to their lower body weight and higher motor activity than Atak-S hens. In an earlier study, a higher percentage of cracked and broken eggs was observed for Atak-S hens (0.44%) than for a white laying hen genotype (Lohmann-LSL) (0.31%) in an organic system [35].

It is known that different commercial laying hen genotypes produce different sizes of eggs, and consequently, the weights and ratios of yolk, albumen, and shell can differ [36]. Our results support findings of Küçükyılmaz et al. [35] of higher yolk ratio in Lohmann-LSL hens and higher albumen ratio in Atak-S hens in organic systems. The increase in yolk ratio and decline in albumen ratio observed with increasing hen age confirm previous findings of Van den Brand et al [31], Rizzi and Chiericato [37], Tůmová and Ledvinka [38], and Zita et al. [39].

Egg exterior characteristics, namely egg shape index, eggshell breaking strength, and shell thickness, are important quality parameters in commercial handling and transport [36,40]. In the present study, egg quality parameters were affected by both genotype and age. A higher shape index was observed for Atak-S eggs, confirming the findings of Küçükyılmaz et al. [35] and Onbaşılar et al. [41]. On the other hand, it was found that egg shape index decreased as hen age increased in both genotypes. Similarly, Onbaşılar et al. [41] found that egg shape index showed a decline from 78.13% at 20 weeks of age to 75.03% at 70 weeks of age. This change could be related to older hens producing longer eggs due to some anatomical structural changes to the shape of the pelvic bones with aging in laying hens [41]. However, Tůmová et al. [42] observed no significant effect of hen genotype on egg shape index. We found higher breaking strength and shell thickness for brown Atak-S eggs, whereas Küçükyılmaz et al. [35] reported higher eggshell breaking strength and thickness for white Lohmann-LSL eggs (4.225 g/cm^2^ and 409.17 μ, respectively) than for brown Atak-S eggs (3.715 g/cm^2^ and 388.56 μ, respectively) in organic systems.

Our study showed that hen genotype affected yolk index, confirming the findings of Zita et al. [39] and Ledvinka et al. [43]. Similar to our results, Leyendecker et al. [44] found a higher Haugh unit for eggs obtained from white hens than for eggs obtained from brown hens. Bozkurt and Tekerli [45], Ledvinka et al. [43], and Kraus and Zita [46] indicated that yolk index decreased with hen age. Our results relating to yolk color support the findings of Ledvinka et al. [43], who indicated that yolk color was significantly affected by hen age, and of Kraus and Zita [46], who found a significant effect of hen genotype on yolk color.

Observed changes in albumen index and Haugh unit by hen genotype and age are similar to the previous findings of Tůmová et al. [38], Zita et al. [39], Bozkurt and Tekerli [45], and Kraus and Zita [46]. Our data indicated a possible relationship between albumen index and Haugh unit. Similarly, Kraus and Zita [46] found that when albumen index increased, Haugh unit also showed an increment. Further, Molnár et al. [47] observed a decline of 0.38 in Haugh unit for each week after 60 weeks of age. However, other studies have found no significant effects of genotype on Haugh unit [37].

The observed changes in protein content of the eggs by genotype could be related to differences in DFI and EP level between Atak-S and Atabey birds. Küçükyılmaz et al. [35] also found a higher protein level in brown eggs than in white eggs. Some studies have found that hen genotype affects yolk fatty acid content [48,49,50]. Our results showed that brown (Atak-S) eggs had a lower content of SFAs than white (Atabey) eggs (*p* < 0.01), confirming the findings of Ayerza and Coates [51]. On the other hand, it is known that the content of n-3 PUFAs in egg yolk can be enhanced in hens consuming fresh vegetables, insects, and worms [52]. The higher content of C18:3n observed in Atabey eggs could be related to more activity and foraging behavior of this genotype compared with Atak-S. Likewise, Hammershøj and Johansen [53] reported that the fatty acid content of egg yolk is largely influenced by the amount of grasses and herbs consumed by the hens in free-range systems.

## 5. Conclusions

In conclusion, the findings in this study are of practical significance for consumers and for Turkish producers employing alternative egg production systems such as free-range and organic production. This study examined the suitability of local brown and white egg genotypes (Atak-S and Atabey) for free-range systems. Atabey hens showed a higher egg production rate with better feed utilization, as well as better yolk, albumen, and eggshell quality, than Atak-S hens. However, the Atak-S hens gave higher egg weight, darker yolk color, stronger shell structure, and a lower level of SFAs in yolk. These findings on genotype effects could help producers in their choices for profitable production and for meeting consumer demands on egg quality and egg yolk fatty acid levels. The findings could contribute to more sustainable poultry production by improving the use of safe and more natural production systems and increase customer satisfaction.

## Figures and Tables

**Table 1:** Composition and nutrient content of phase I (19–40 weeks) and phase II (41–72 weeks) laying hen diets.

Item	Weeks	
	19–40	41–72
Ingredient (g/kg)		
Corn, grain	262	278
Wheat	323	332
Soybean meal, 48%	165	140
Sunflower meal, 32%	35	35
Milled alfalfa	88	88
Soybean oil	30	30
Sodium chloride	2	2
Limestone	80	80
Dicalcium phosphate	10	10
Premix *	5	5
Calculated chemical analysis		
ME, kcal/kg	2750	2800
Available phosphorus	0.48	0.46
Chemical analysis		
Dry matter	90.5	90.9
Crude ash	7.2	7.2
Crude fiber	5.1	5.9
Crude protein	17.86	16.45
Calcium	3.50	3.50
Phosphorus	0.73	0.71

* Composition of premix (1 kg): vitamin A, 8.000 IU; vitamin D3, 2.000 IU; vitamin B2, 4 mg; vitamin B12, 10 mg; vitamin E, 15 mg; vitamin K3, 2 mg; vitamin B1, 3 mg; niacin, 30 mg; cal-D-pantothenic acid, 10 mg; vitamin B6, 5 mg; folic acid, 1 mg; D-biotin, 0.05 mg; vitamin C, 50 mg; choline chloride, 300 mg; Mn, 60 mg; Zn, 50 mg; Fe, 60 mg; Cu, 5 mg; Co, 0.5 mg; iodine, 2 mg; Se, 0.15 mg.

**Table 2:** Mean egg production values and mean egg weight for two Turkish laying hen genotypes (Atak-S and Atabey) in a free-range system.

Main Factors	Hen-Day Egg Production (%)	Hen-House Egg Production (%)	Egg Weight (g)
Genotype			
Atak-S (brown)	74.9 ^b^	70.3 ^b^	62.6 ^a^
Atabey (white)	78.1 ^a^	75.9 ^a^	60.2 ^b^
SEM	0.34	0.46	0.11
Age (weeks)			
19–24	48.4 ^h^	47.8 ^h^	52.5 ^h^
25–30	89.4 ^a^	87.7 ^a^	57.6 ^g^
31–36	85.6 ^b^	83.5 ^b^	60.4 ^f^
37–42	82.4 ^c^	79.5 ^c^	62.1 ^e^
43–48	80.0 ^d^	76.4 ^cd^	62.9 ^de^
49–54	78.3 ^de^	74.2 ^de^	63.3 ^cd^
55–60	76.7 ^ef^	71.9 ^ef^	64.1 ^bc^
61–66	75.3 ^f^	70.3 ^f^	64.5 ^ab^
67–72	72.2 ^g^	66.6 ^g^	65.2 ^a^
SEM	0.72	0.98	0.24
Genotype × Age			
Atak-S × 19–24	56.4 ^k^	55.4 ^j^	55.8 ^i^
Atak-S × 25–30	88.7 ^ab^	86.2 ^a^	58.6 ^h^
Atak-S × 31–36	81.7 ^de^	78.4 ^cd^	61.9 ^g^
Atak-S × 37–42	78.8 ^efg^	74.5 ^def^	62.7 ^efg^
Atak-S × 43–48	77.2 ^fgh^	72.2 ^efg^	63.7 ^cde^
Atak-S × 49–54	75.8 ^ghi^	70.2 ^fgh^	64.1 ^cd^
Atak-S × 55–60	74.6 ^hi^	68.4 ^gh^	64.9 ^abc^
Atak-S × 61–66	72.9 ^i^	66.5 ^h^	65.4 ^ab^
Atak-S × 67–72	67.8 ^j^	60.9 ^i^	66.1 ^a^
Atabey × 19–24	40.4 ^i^	40.2 ^k^	49.2 ^j^
Atabey × 25–30	90.1 ^a^	89.2 ^a^	56.6 ^i^
Atabey × 31–36	89.4 ^ab^	88.6 ^a^	59.0 ^j^
Atabey × 37–42	86.0 ^bc^	84.4 ^ab^	61.5 ^g^
Atabey × 43–48	82.9 ^cd^	80.5 ^bc^	62.1 ^fg^
Atabey × 49–54	80.7 ^def^	78.1 ^cd^	62.5 ^efg^
Atabey × 55–60	78.7 ^efg^	75.4 ^cde^	63.2 ^def^
Atabey × 61–66	77.7 ^fgh^	74.1 ^def^	63.6 ^de^
Atabey × 67–72	76.6 ^ghi^	72.3 ^efg^	64.3 ^bcd^
SEM	1.02	1.39	0.33
*p*-values			
Genotype	<0.01	<0.01	<0.01
Age	<0.01	<0.01	<0.01
Genotype × Age	<0.01	<0.01	<0.01

*n* = 3 replicates per treatment group (70 laying hens/pen). ^a–k^ Means in the same column with different letters differ significantly (*p* < 0.05).

**Table 3:** Mean body weight, daily feed intake, and feed conversion rate (FCR) for two Turkish laying hen genotypes (Atak-S and Atabey) in a free-range system.

Main Factors	Body Weight (g)	Daily Feed Intake (g)	FCR
Genotype			
Atak-S (brown)	2087.3 ^a^	117.2 ^a^	2.54 ^a^
Atabey (white)	1497.4 ^b^	109.8 ^b^	2.48 ^b^
SEM	5.94	0.40	0.01
Age (weeks)			
19–24	1529.2 ^h^	100.3 ^g^	4.05 ^a^
25–30	1630.7 ^g^	103.7 ^f^	2.02 ^h^
31–36	1679.8 ^f^	109.2 ^e^	2.12 ^g^
37–42	1744.0 ^e^	112.7 ^d^	2.21 ^f^
43–48	1831.0 ^d^	116.5 ^c^	2.32 ^e^
49–54	1874.2 ^c^	117.7 ^c^	2.38 ^de^
55–60	1927.7 ^b^	118.8 ^bc^	2.42 ^d^
61–66	1943.7 ^ab^	121.0 ^ab^	2.50 ^c^
67–72	1971.3 ^a^	121.8 ^a^	2.60 ^b^
SEM	12.59	0.85	0.03
Genotype × Age			
Atak-S × 19–24	1753.3 ^e^	108.0 ^hi^	3.43 ^b^
Atak-S × 25–30	1896.0 ^d^	111.0 ^fgh^	2.14 ^j^
Atak-S × 31–36	1924.0 ^d^	113.3 ^efg^	2.24 ^i^
Atak-S × 37–42	2016.3 ^c^	116.3 ^bcde^	2.35 ^g^
Atak-S × 43–48	2132.7 ^b^	118.7 ^bcd^	2.41 ^ef^
Atak-S × 49–54	2183.3 ^b^	119.7 ^abc^	2.46 ^e^
Atak-S × 55–60	2276.0 ^a^	120.7 ^ab^	2.49 ^e^
Atak-S × 61–66	2287.7 ^a^	123.3 ^a^	2.59 ^d^
Atak-S × 67–72	2316.7 ^a^	124.0 ^a^	2.77 ^c^
Atabey × 19–24	1305.0 ^j^	92.7 ^j^	4.66 ^a^
Atabey × 25–30	1365.3 ^j^	96.3 ^j^	1.89 ^i^
Atabey × 31–36	1435.7 ^i^	105.0 ^i^	1.99 ^k^
Atabey × 37–42	1471.7 ^hi^	109.0 ^ghı^	2.06 ^jk^
Atabey × 43–48	1529.3 ^gh^	114.3 ^def^	2.22 ^i^
Atabey × 49–54	1565.0 ^fg^	115.7 ^cde^	2.29 ^h^
Atabey × 55–60	1579.3 ^f^	117.0 ^bcde^	2.35 ^g^
Atabey × 61–66	1599.7 ^f^	118.7 ^bcd^	2.40 ^f^
Atabey × 67–72	1626.0 ^f^	119.7 ^abc^	2.43 ^ef^
*p*-values	17.81	1.20	0.04
Genotype	<0.01	<0.01	<0.01
Age	<0.01	<0.01	<0.01
Genotype × Age	<0.01	<0.01	<0.01

*n* = 3 replicates per treatment group (70 laying hens/pen). ^a–k^ Means in the same column with different letters differ significantly (*p* < 0.05).

**Table 4:** Mean egg weight and egg content for two Turkish laying hen genotypes (Atak-S and Atabey) in a free-range system.

Main Factors	Egg Weight (g)	Yolk Ratio (%)	Albumen Ratio (%)	Shell Ratio (%)
Genotype				
Atak-S (brown)	61.1 ^a^	21.6 ^b^	67.1	11.3 ^a^
Atabey (white)	58.9 ^b^	22.1 ^a^	66.9	11.0 ^b^
SEM	2.2	0.14	0.16	0.09
Age (weeks)				
24	51.9 ^d^	19.5 ^c^	69.1 ^a^	11.4
40	61.0 ^c^	22.0 ^b^	66.7 ^b^	11.3
56	62.9 ^b^	22.7 ^a^	66.2 ^b^	11.1
72	64.1 ^a^	22.8 ^a^	66.1 ^b^	11.1
SEM	2.2	0.14	0.16	0.09
Genotype × Age				
Atak-S × 24	54.3 ^e^	19.7 ^c^	68.9 ^a^	11.4
Atak-S × 40	61.9 ^c^	21.5 ^b^	67.0 ^b^	11.5
Atak-S × 56	63.5 ^b^	22.4 ^a^	66.5 ^b^	11.1
Atak-S × 72	64.8 ^a^	22.5 ^a^	66.4 ^b^	11.1
Atabey × 24	49.6 ^f^	19.4 ^c^	69.6 ^a^	11.0
Atabey × 40	60.2 ^d^	22.4 ^a^	66.5 ^b^	11.1
Atabey × 56	62.2 ^c^	23.0 ^a^	66.1 ^b^	10.9
Atabey × 72	63.4 ^b^	23.0 ^a^	66.1 ^b^	10.9
SEM	2.8	0.29	0.32	0.18
*p*-values				
Genotype	<0.01	0.004	0.496	0.001
Age	<0.01	<0.01	<0.01	0.083

*n* = 30 eggs/genotypes/age. ^a–f^ Means in the same column with different letters differ significantly (*p* < 0.05).

**Table 5:** Mean values of exterior egg quality parameters for two Turkish laying hen genotypes (Atak-S and Atabey) in a free-range system.

Main Factors	Shape Index (%)	Breaking Strength (g/cm^2^)	Shell Thickness (mm)
Genotype			
Atak-S (brown)	77.9 ^a^	3.429 ^a^	0.371 ^a^
Atabey (white)	76.0 ^b^	2.982 ^b^	0.361 ^b^
SEM	0.5	0.03	0.001
Age (weeks)			
24	78.0 ^a^	3.398 ^a^	0.378 ^ab^
40	76.5 ^b^	3.320 ^ab^	0.384 ^a^
56	76.7 ^b^	3.215 ^b^	0.371 ^b^
72	76.7 ^b^	2.890 ^c^	0.333 ^c^
SEM	0.5	0.03	0.001
Genotype × Age			
Atak-S × 24	79.7 ^a^	3.613	0.381 ^ab^
Atak-S × 40	77.7 ^ab^	3.557	0.387 ^a^
Atak-S × 56	77.3 ^bc^	3.480	0.372 ^b^
Atak-S × 72	77.0 ^bc^	3.067	0.347 ^c^
Atabey × 24	76.3 ^bc^	3.183	0.375 ^ab^
Atabey × 40	75.3 ^c^	3.083	0.381 ^ab^
Atabey × 56	76.0 ^bc^	2.950	0.370 ^b^
Atabey × 72	76.3 ^bc^	2.713	0.320 ^d^
SEM	0.6	0.06	0.004
*p*-values			
Genotype	<0.01	<0.01	<0.01
Age	0.005	<0.01	<0.01
Genotype × Age	0.027	0.294	<0.01

*n* = 30 eggs/genotype/age. ^a–d^ Means in the same column with different letters differ significantly (*p* < 0.05).

**Table 6:** Mean values of interior egg quality parameters in two Turkish laying hen genotypes (Atak-S and Atabey) in a free-range system.

Main Factors	Yolk Index (%)	Yolk Color	Albumen Index (%)	Haugh Unit
Genotype				
Atak-S (brown)	43.1 ^b^	12.9 ^a^	10.9 ^b^	88.5 ^b^
Atabey (white)	44.4 ^a^	12.2 ^b^	12.3 ^a^	92.4 ^a^
SEM	0.6	0.2	0.3	0.8
Age (weeks)				
24	41.8 ^b^	12.3 ^ab^	11.8 ^ab^	87.1 ^c^
40	42.7 ^b^	12.8 ^a^	12.9 ^a^	94.2 ^a^
56	46.9 ^a^	12.8 ^a^	11.4 ^bc^	91.4 ^ab^
72	43.7 ^b^	12.2 ^b^	10.2 ^c^	89.2 ^bc^
SEM	0.6	0.2	0.3	0.8
Genotype × Age				
Atak-S × 24	41.0	13.0	11.4	86.5
Atak-S × 40	42.3	13.3	11.9	91.9
Atak-S × 56	46.3	13.0	10.8	88.7
Atak-S × 72	42.9	12.3	9.5	87.0
Atabey × 24	42.6	11.7	12.2	87.7
Atabey × 40	43.2	12.3	13.9	96.4
Atabey × 56	47.4	12.7	12.1	94.1
Atabey × 72	44.5	12.0	11.0	91.4
SEM	1.1	0.3	0.6	1.7
*p*-values				
Genotype	0.026	<0.01	<0.01	<0.01
Age	<0.01	0.016	<0.01	<0.01
Genotype× Age	0.969	0.123	0.624	0.307

*n* = 30 eggs/genotype/age. ^a–c^ Means in the same column with different letters differ significantly (*p* < 0.05).

**Table 7:** Chemical composition and yolk fatty acid profile in eggs from two Turkish laying hen genotypes (Atak-S and Atabey) in a free-range system (56 weeks of age).

Parameters	Genotype		SEM	*p*-Value
	Atak-S (Brown)	Atabey (White)		
Albumen				
Dry matter (%)	86.6	88.2	0.8	0.009
Ash (%)	0.59	0.55	0.04	0.137
Protein (%)	13.6	11.9	0.9	0.009
Fat (%)	0.12	0.14	0.02	0.347
Yolk				
Dry matter (%)	58.9	56.8	1.8	0.065
Ash (%)	1.4	1.3	0.2	0.145
Protein (%)	4.6	5.2	0.4	0.031
Fat (%)	57.8	57.5	1.2	0.711
Fatty acids (% of total fatty acids)				
Saturated fatty acids (SFAs)				
Myristic acid (C14:0)	0.31	0.38	0.03	<0.01
Pentadecanoic acid (C15:0)	0.07	0.06	0.02	0.139
Palmitic acid (C16:0)	23.3	25.5	0.21	<0.01
Margaric acid (C17:0)	0.21	0.05	0.01	<0.01
Stearic acid (C18:0)	8.13	9.23	0.32	<0.01
Arachidic acid (C20:0)	0.11	0.15	0.01	<0.01
Monounsaturated fatty acids (MUFAs)				
Palmitoleic acid (C16:1)	1.82	2.30	0.16	<0.01
Heptadedenoseic acid (C17:1)	0.07	0.04	0.03	0.008
Oleic acid (C18:1c)	37.5	37.1	1.41	0.461
Eicosenoic acid (C20:1)	0.24	0.27	0.01	0.002
Erucic acid (C22:1)	2.25	2.11	0.07	<0.01
Nervonic acid (C24:1)	0.21	0.15	0.01	<0.01
Polyunsaturated fatty acids (PUFAs)				
Linoleic acid (C18:2c)	18.00	18.30	0.54	0.184
Alphalinolenic acid (C18:3n)	0.39	0.43	0.03	0.001
Eicosadienoic acid (C20:2)	0.18	0.21	0.01	<0.01
Eicosatrienoic acid (C20:3n6)	0.13	0.14	0.05	0.516
Docosahexaenoic acid (C22:6)	1.10	0.80	0.09	<0.01
SFAs	32.1	35.4	0.51	<0.01
Unsaturated fatty acids (UFAs)	61.9	61.9	1.35	0.991
MUFAs	42.1	42.0	1.25	0.796
PUFAs	19.8	19.9	0.56	0.566
UFAs/SFAs	1.93	1.74	0.04	<0.01
MUFAs/SFAs	1.31	1.18	0.03	<0.01
PUFAs/SFAs	0.62	0.56	0.02	<0.01

*n* = 12 eggs/genotype.

## Data Availability

All data sets collected and analyzed during the current study are available from the corresponding author on fair request.
